# Clinical Profile and Predictors of Outcome in Spontaneous Intracerebral Hemorrhage from a Tertiary Care Centre in South India

**DOI:** 10.1155/2020/2192709

**Published:** 2020-01-27

**Authors:** Ajay Hegde, Girish Menon, Vinod Kumar, G. Lakshmi Prasad, Lakshman I. Kongwad, Rajesh Nair, Raghavendra Nayak

**Affiliations:** ^1^Department of Neurosurgery, Kasturba Medical College, Manipal Academy of Higher Education, Manipal, India; ^2^Institute of Neurological Sciences, NHS Greater Glasgow and Clyde, UK

## Abstract

*Background. *This article attempts to evaluate the clinical profile and outcome determinants following hypertensive SICH in a South Indian population. The study represents the largest series of SICH reported from a single centre in India. *Materials and Methods.* Prospective data collection and analysis of patients with SICH admitted to our centre between 1^st^ January 2015 and 31^st^ December 2018. The variables analysed include: age, sex, comorbidities, Glasgow coma score (GCS) on admission, radiological features, treatment modality, and outcome at three months. Modified Rankin score (mRS) was used to assess the outcome at discharge and three months. *Results.* Our study group of 905 patients included 638 males and 267 females and the mean age at presentation was 58.10 ± 12.76 years. The study group included 523 patients (57.8%) previously diagnosed hypertensive, of whom 36.3% (*n* = 190) were on irregular medication. The most frequent locations of hematoma were basal ganglia (478), thalamus (202), lobar (106), cerebellar (61), brainstem (31), and primary intraventricular haemorrhage (27). Secondary intraventricular extension was seen in 425 (47%) patients on admission. The mean volume of the clot on admission was 23.45 ± 19.79 ml, and clot progression was seen in only 46 (5.08%) cases. Surgical evacuation through craniotomy was done in 147 (16.8%) patients, and external ventricular drainage (EVD) was placed in 56 (6.2%) patients. Overall 3−month mortality was 30.1% (266 patients). On the last follow up a favorable outcome (mRS 0−3) was observed in 412 (45.53%) patients and a poor outcome (mRS 4−5) in 207 patients (22.87%). Independent predictors of mortality are Age >70 (*p* = <0.001, OR 4.806, 95% CI 3.064–7.54), admission GCS <8 (*p* = <0.001, OR7.684, 95% CI 5.055#x2013;11.68), and Hematoma volume >30 ml (*p* = <0.001, OR 2.45, 95% CI 1.626–3.691). Intraventricular haemorrhage was an additional poor outcome predictor (*p* < 0.015, CI 1.105–2.585). Surgical evacuation reduced mortality in the group, but morbidity rates remained the same. *Conclusions.* SICH predominantly affects a younger population in India in comparison to the Western society. Elderly age, poor GCS on admission, clot volume above 30 ml and intraventricular extension remain the most consistent predictors of death and poor outcome. Further studies are needed to assess the risk of SICH among hypertensive patients and to prognosticate the outcome after SICH using novel predictors, including biomarkers.

## 1. Introduction

Spontaneous Intracerebral Haemorrhage (SICH) is the second most common cause of stroke and accounts for 7.5−30% of all strokes [1, 2]. Haemorrhagic stroke is generally associated with higher morbidity and mortality rates than ischemic stroke [3–5]. Only one-fifth of the patients regain functional independence after SICH and between one-fourth to half of the patients succumb to the bleed [1, 6]. Optimal management is controversial, and considerable debate exists primarily on the role of surgery in SICH [7]. In developing countries like India with severe resource constraints, treatment strategies need to be customised given the high morbidity and mortality associated with SICH. Published literature from India on outcome following SICH is limited [8–18]. The goal of this single institution based study was to analyse the clinical profile, to assess the functional outcome and to identify the outcome prognosticators after SICH in a tertiary care hospital located in a coastal town in South India.

## 2. Materials and Methods

The study was approved by the Institute Ethics Committee, Manipal University (Approval No **IEC 209/2015)**. All patients above the age of 18 years who were presented to the Emergency Department between 1^st^ January 2015 and 31^st^ December 2018 with computerised tomography (CT) evidence of SICH were included in the study. Patients with post-traumatic hematomas, intracranial space-occupying lesions with bleeds, haemorrhagic transformation of an ischemic stroke, vascular malformations, and aneurysms were excluded from the study. Demographic data, comorbidities, clinical, radiological data, and information about surgical interventions were recorded in an online registry. Volume of hematoma was measured using the axbxc/2 method [19]. Hematoma expansion was determined if sequential brain imaging was available, and defined as a relative parenchymal volume increase of more than 33% from initial to follow‐up imaging within 3–72 hours [20]. All patients were admitted to an exclusive neurosurgical intensive care unit and started on antihypertensives (Amlodipine, Labetalol) and antiedema measures (Mannitol 20%). Patients with lobar hematoma and patients planned for surgery were started on antiepileptics (Phenytoin sodium 7 mg/kg in divided doses). All patients had a follow-up CT scan at 24 hours of admission or on clinical deterioration, whichever was earlier. Surgical evacuation was offered to all patients with supratentorial hematoma volume >30 ml or midline shift of >1 cm. Posterior fossa cerebellar hematomas with maximum diameter >3 cm were offered surgical intervention. Thalamic or Basal Ganglia bleeds with intraventricular extension and significant hydrocephalus with a GCS<8 were managed with an External Ventricular Drain (EVD) followed by administration of Inj. Streptokinase 30,000 IU daily through the EVD. The EVD was retained for a maximum of five days following its insertion. The outcome determinants were mortality and morbidity as measured using the modified Rankin Scale (mRS) at discharge and three months. A mRS of 4 and 5 were considered as poor outcome and mRS of 0−3 as good outcome. Ninety day mRS was recorded in the outpatient clinic. In cases where the patient failed to visit the clinic, a telephonic mRS was recorded.

### 2.1. Statistical Methods

Statistical analyses were performed with SPSS 24.0. Descriptive statistics including mean, median and standard deviation were computed for baseline characteristics. Chi-Square test was used to compare categorical variables, and Student t-test and Mann Whitney test was applied to calculate the *p*-value for continuous variables for univariate statistics. Wilcoxon signed-rank test was used to compare mRS at discharge and 90 days. Predictors of poor outcome and death at three months were analyzed using logistic regression analysis. Variables with a *p*-value less than 0.05 at univariate level were considered significant.

## 3. Results

In total, 1052 patients with spontaneous ICH requiring hospitalization were identified within the study period. Based on our inclusion and exclusion criteria, 905 patients were included in the study. Twenty patients were lost to follow up, and their mRS at 90 days could not be obtained.

### 3.1. Patient Demographics

The mean age of our study group was 58.10 ± 12.76 (18 years to 93 years), and 638 (70.5%) of them were males. Nearly one-third of the patients (266 patients 29.39%) were below the age of 50 years.

### 3.2. Risk Factors

History of arterial hypertension was present in 57.8% (*n* = 523) of patients. Of these 523 hypertensive patients, nearly one-third 36.3% (*n* = 190) were on irregular treatment, and their mean duration of hypertension was 5.23 ± 4.64 years. The remaining 333 patients were newly diagnosed hypertensive on presentation. A definitive history of diabetes was available for 245 (27.1%) patients with a mean duration of 7.1 years. The mean random blood glucose levels on admission were 162.49 ± 69.05 dl. At the time of the ICH, a total of 83 (9.2%) patients were on antiplatelet medications (Table 1).

### 3.3. Clinical Features

The most common presenting symptom was sudden onset headache (62%), contralateral weakness (57%) with a drop in sensorium. The median GCS on admission was 12 (IQR 8–14) and 233 patients presented with a GCS of <8. Mean systolic blood pressure on admission was 175.09 ± 59.08 mmHg and diastolic blood pressure was 99.49 ± 14.02 mmHg.

### 3.4. Imaging Characteristics

The mean hematoma volume in supratentorial bleeds was 23.45 ± 19.79 ml. Intraventricular extension of hematoma was noticed in 425 (47%) of patients. Only 46 patients (5.08%) had an increase in the size of hematoma on follow-up imaging. The most common location of the haemorrhage was capsuloganglionic (52.81%, *n* = 478), thalamic (22.32%, *n* = 202) followed by lobar (11.71%, *n* = 106), cerebellar (6.7%, *n* = 61), brainstem (3.4%, *n* = 31), and primary intraventricular haemorrhage (2.9%, *n* = 27) (Table 2). Thalamic and cerebellar hematomas were found in older age group in comparison to basal ganglia, lobar, and primary intraventricular haemorrhages. Intraventricular extension and hydrocephalus were more common with thalamic bleeds. Hematoma expansion was more common with basal ganglionic bleeds (35/478) (Tables 1, 2). ICH score was computed for all patients on admission, and the distribution is shown in Table 3.

### 3.5. Surgery

Surgical evacuation of the clot through craniotomy was performed in 147 (16.8%) patients. External ventricular drainage for intraventricular haemorrhage was performed in 56 (6.2%) patients.

### 3.6. Outcome

The overall mortality was 30.1% (266 patients). Of these, 116 (12.81%) patients succumbed during initial hospitalization, and 150 (16.57%) patients died within three months of follow up. For survivors at three months, the median mRS was 4 (IQR 3−6). Functional status at discharge and three months are presented in Table 4. Among 639 survivors at three months, 207 (33.4%) had a mRS 4-5 (poor outcome), and 412 had a favourable outcome (mRS 0−3), and twenty patients were lost to follow up. We observed that following discharge, many of the patients showed a gradual recovery with rehabilitative measures. The number of patients with favourable outcome ( mRS <=3) increased from 196 to 412 at the end of 3 months. The number of patients with poor outcome (mRS 4-5) dropped to 207 at the end of 3 months (Table 4). Wilcoxon Signed Rank Test showed this recovery pattern to be statistically significant (*p* = <0.001) (Table 4).

### 3.7. Prognostic Factors

Summary of the analysis of the various factors related to mortality and outcome are shown in Table 1. Step wise Binary logistic regression of significant factors on univariate analysis was performed. Age > 70 years, GCS < 8, Volume > 30 ml, presence of hydrocephalus, and hematoma growth were significant factors predicting mortality. Similarly the factors predicting poor outcome (mRS 4-5) were Age > 70 years, GCS < 8, Volume > 30 ml, and intraventricular extension. The summary of the above parameters with their significance and Confidence interval are tabulated in (Table 5).

## 4. Discussion

Hemorrhagic stroke has devastating consequences. The need to identify potential risk factors, initiate corrective measures, and customize treatment cannot be overemphasized, especially in resources limited setting as in India. Published epidemiological studies related to hemorrhagic stroke from India are sparse. [8–18]. This study group comprising of 905 patients with spontaneous intracerebral hematoma is the largest single-institution series from India. (Table 1).

### 4.1. Age and Sex

Primary SICH is considered to be a disease of the elderly. The mean age of the study group in Hemphill et al.'s landmark paper was 66 years [21]. The mean age of patients in our study was 58.10 ± 12.76, and twenty-seven patients (2.9%) were above 80 years in our group. This comparatively younger age of incidence has been reported uniformly across India and appears to be a characteristic feature of the SICH in the Indian subcontinent [9, 12, 22, 23]. Increasing age is also associated with increased morbidity and elderly population above 70 years had a high mortality rate (46.1%) in our series. Several authors have shown that older adults with acute ICH experienced the worse outcomes compared with their younger counterparts, including death, dependency, and overall quality of life [5, 24–26].

### 4.2. Hypertension

Hypertension is the most common risk factor in all the studies relating to SICH. Feldmann et al. have reported a relative risk of 3.9 for ICH in patients with hypertension [27]. A definite history of hypertension could be elicited in only 57.8% of our patients, and nearly third of these patients were noncompliant hypertensive on irregular medication. The remaining patients had never under gone a prior medical evaluation and were diagnosed to have raised blood pressure on presentation. This observation is in contrast to the higher rates of preexisting hypertension (70−80%) observed in other Indian studies [9, 12, 22]. The adverse effect of high blood pressure is assumed to be through hematoma expansion, which was, however, observed only in 5.08% patients in our series. This increase in hematoma volume did not correlate with mortality or outcome in our series (*p* = 1.227). We do follow a policy of aggressive blood pressure reduction as it has been well proven that aggressive BP lowering is beneficial in reducing haematoma growth, however it seldom translates to the better clinical outcome [28].

### 4.3. Diabetes and Hyperglycemia

It is proposed that high blood glucose at admission contributes to poor outcome, due to exacerbation of cerebral oedema and cerebral damage. A recent meta-analysis by Zheng et al. in 2018, concluded that hyperglycemia was associated with poor functional outcome in patients with ICH [29]. However, the pool of available evidence about blood glucose variability and ICH is still limited, and random blood glucose has not been a predictor of mortality in the Indian ICH studies. [9, 12, 30] In our series, multivariate logistic regression analysis failed to demonstrate an association between blood glucose and poor outcome.

### 4.4. Glasgow Coma Score

One of the most consistent predictors of poor outcome, in almost all published series, has been a Glasgow Coma Score of less than 8. Indian studies to have reported similar findings with Bhatia et al. and Namani et al. reporting a fatality of 72.9% and 100% respectively with poor GCS on admission [9, 22]. We observed a 66.5% mortality in patients with an admission of GCS <8 and a lower GCS was a statistically significant predictor of poor outcome both on univariate and multivariate analysis.

### 4.5. Clot Volume

Volume of the clot is also a crucial radiological predictor of outcome [2]. It has been reported that each mL increase in baseline clot volume is associated with a 1% increased risk of mortality [31]. It has also been shown that clinically significant hematoma growth occurs in up to 1/4 of ICH patients [32] and for each 10% increase in hematoma volume growth, the risk of death increases by 5% [31]. The mean volume of the clot in the mortality group was 32.45 ± 24.39 ml, and the mean volume amongst the survivors was 17.55 ± 15.59 ml (*p* < 0.001). We dichotomized our supratentorial bleed patients into two groups of volume of 30 ml and observed that 125/234 (53.6%) with clot volume >30 ml were dead at three months. Hematoma expansion was seen in 46 (5.08%) patients in our study. The mean duration of time of onset to the first CT scan in these 46 patients was 6 : 20 hours (2–17 : 15 hours).The rate of expansion is lesser than other reported studies [28]. The precise reason for the low incidence of clot expansion in our series is not clear. The probable reasons could be relatively younger age at presentation, intensive blood pressure reduction measures, no patients with anticoagulants and lower incidence of patients on antiplatelet medications.

### 4.6. Site of Hematoma

Infratentorial hematomas, especially brain stem hematomas, are known to carry a poor outcome compared to supratentorial hematoma. In our series, 17 of the 31 patients with brain stem hematoma expired, and the median mRS at 90 days for the remaining 14 patients was 6. Of the 61 patients with cerebellar hematoma 19 expired and the median mRS at 90 days for the remaining 42 patients was 3. In the INTERACT 2 trial, of the 2,066 patients included in the analyses, the involvement of posterior limb of internal capsule and thalamus and infratentorial sites increased risks of death or major disability [33]. In our series of 780 patients with supratentorial clots, lobar hematomas had the best outcome (*p* < 0.001).

### 4.7. Intraventricular Extension

Intraventricular extension (IVE) is seen in around 40–60% of patients with spontaneous ICH and is known to be a significant predictor of 30-day mortality and long term outcome [9, 12, 21]. Subgroup analysis from the STICH 1 Trial data concluded that the absence of IVH resulted in better outcomes. (31.4% vs. 15.1%; *p* < 0.001) and the presence of hydrocephalus lowered the likelihood of favourable outcome still further to 11.5% (*p* = 0.031) [7]. Delayed Intraventricular haemorrhage (dIVH) too is known to adversely affect the outcome. In the INTERACT 2 study, dIVH had greater odds of 90-day death or major disability versus initial IVH [34]. In our cohort, 425 patients (47%) had IVE of hematoma, and IVH was an independent predictor of the poor outcome (*p* = 0.015). Hydrocephalus was observed in 236 patients (26.1%) in our group and was also an independent predictor of mortality (*p* = 0.014). This probably can be attributed to the significant number of small to moderate thalamic bleeds with IVE in our study.

### 4.8. Surgery

Role of surgery in SICH remains controversial. A meta-analysis published by Prasad et al. after the STICH 1 trail concluded that surgery added to medical management reduces the odds of being dead or dependent compared with medical management alone [35]. In Troberg's, study supposedly the most extensive study on long-term functional outcome with the longest follow-up (up to 10.8 years) of survival and functional status after surgery for ICH, 31% of all operated ICH patients were deceased after 1 year and only 24% of patients available for assessment of long-term functional outcome were independent in activities of daily life [33]. Preictal heart disease and reduced level of consciousness before surgery were the most consistent predictors of mortality regardless of follow-up time. Our study, however, showed no significant influence of clot evacuation on mortality (*p* = 0.621).

### 4.9. ICH Scoring

Several scores for predicting functional outcome and mortality after ICH have been developed. The ICH score proposed by Hemphill has stood the test of time for prognosticating SICH [8]. For SICH scores of 0, 1, 2, 3, 4, and 5 our mortality rates were 7%, 16%, 33%, 62%, 95%, and 50% in comparison to Hemphill et al. 0%, 13%, 26%, 72%, 94%, and 100%. The Essen ICH score is determined only by clinical variables (age, the severity of neurological deficits, and level of consciousness) and has the advantage of not requiring the measurement of ICH volume [36]. The FUNC score additionally takes into consideration the occurrence of preICH cognitive impairment [37]. More recently, scores like the BAT score have been designed for identifying predictors of hematoma expansion [38]. We prefer to use the ICH score due to its ease in application. In an earlier publication, Hegde et al. attempted to validate the ICH score in an Indian setting and suggested reducing the age cut off from 80 years to 70 years in the original SICH score [18]. This was influenced by the fact that the mean age of the affected group in our study as well as in other Indian studies is much younger compared to the Western population. However, we do agree with Pinho et al. that even though the use of prognostic scores is recommended other factors must also be weigh when evaluating individual patients and an early subjective clinical judgment by experienced clinicians is not inferior to the application of formal prognostic scores in predicting outcome [24, 39].

## 5. Morbidity and Mortality

Less than half of the patients with ICH survive one year, and less than a third survive five years [40]. Poon et al.'s systematic review and meta-analysis of 122 longitudinal cohort studies reporting long-term (>30 days) outcome after spontaneous ‘primary' ICH have shown a 1-year survival of 46.0% and a 5-year survival of 29.2% following SICH [31]. The 1-month case fatality after ICH has remained unchanged for several decades at around 40%, but the outcome in the longer term is less clear [5]. In addition to the risk of mortality and poor functional outcome, survivors are also at a considerable risk of recurrent ICH and vaso occlusive events, including ischemic strokes. Three of our patients presented again with SICH at other sites and two developed ischemic infarcts. Our mortality rate at the end of 3 months was 30.1%, which is marginally better than other quoted Indian studies [9, 12]. With a median mRS score of 4 at 90 days follow up our overall outcome too appears to be comparable to other reported Indian studies.

## 6. Limitations

The major limitations of our study are short follow up (90 days) and the fact that the outcome assessment is restricted to mRS. Other important parameters, including cognitive disability etc. have not been assessed. Biomarkers and other novel predictors like neurotophil—lymphocyte ratio was not evaluated.

## 7. Conclusion

Spontaneous Intracerebral Hemorrhage predominantly affects a younger population in India in comparison to the Western population. Age > 70 years, GCS < 8 on admission, clot volume above 30 ml, and intraventricular extension and hematoma growth and hydrocephalus remain the most consistent predictors of death and poor outcome following spontaneous intracerebral hematoma. Further studies are needed to assess the risk of SICH among hypertensive patients and to prognosticate outcome after SICH using novel predictors, including bio markers.

## Figures and Tables

**Table 1:** Demographic, etiological, clinical and radiological characteristics of patients with outcome and mortality. GCS—glasgow coma Score, ICH—intracerebral hemorrhage, IVE—intraventricular haemorrhage, EVD—external ventricular drain.

	*n* = 905	Alive (619)	Dead (266)	*p*	Good outcome (412)	Poor outcome (207)	*p*
*Demographics*							
Age (years)	58.10 ± 12.76	56.65 ± 12.33	61.64 ± 13.17	<0.001	56 ± 12.25	57.94 ± 12.42	0.06
*Sex*							
Male	638 (70.5%)	435 (69.5%)	191 (30.5%)	0.687	284 (65.3%)	151 (34.7%)	0.351
Female	267 (29.5%)	184 (71%)	75 (29%)		128 (69.6%)	56 (30.4%)	
*Etiology*							
Hypertension	523 (57.7%)	350 (68.2%)	163 (31.8%)	0.207	226 (64.6%)	124 (35.4%)	0.264
Irregular treatment	190 (21%)	124 (66.3%)	63 (33.7%)	0.346	86 (69.4%)	38 (30.6%)	0.111
Duration	5.23 ± 4.64	5.16 ± 4.89	5.58 ± 4.18	0.479	4.87 ± 5.3	5.7 ± 3.8	0.251
Diabetics	245 (27.1%)	170 (71.7%)	67 (28.3%)	0.508	113 (66.5%)	57 (33.5%)	1
Duration	7.18 ± 4.69	6.76 ± 4.17	7.92 ± 5.,84	0.308	6.24 ± 4.05	7.50 ± 4.32	0.202
Smoking	134 (16.9%)	87 (66.4%)	44 (33.6%)	0.122	47 (54%)	40 (46%)	0.001
Alcohol	276 (30.5%)	193 (71.2%)	78 (28.8%)	0.803	119 (61.7%)	74 (38.3%)	0.068
Previous stroke	84 (9.3%)	51 (62.2%)	31 (37.8%)	0.128	31 (60.8%)	20 (39.2%)	0.355
On antiplatelets	83 (9.2%)	46 (56.8%)	35 (43.2%)	0.007	30 (65.2%)	16 (34.8%)	0.871
*Clinical features*							
Heart rate (bpm)	82.19 ± 16.44	80.36 ± 15.34	86.21 ± 17.91	<0.001	80.03 ± 15.55	81.02 ± 14.93	0.449
Systolic BP (mmHg)	175.09 ± 59.08	172.77 ± 67.82	180.41 ± 32.71	0.08	172.94 ± 80.95	172.42 ± 26.94	0.928
Diastolic BP(mmHg)	99.49 ± 14.02	98.81 ± 13.44	101.07 ± 15.44	0.029	98.20 ± 13.6	100.01 ± 13.08	0.115
GCS (median)	12 (8–14)			<0.001			
GCS <8	233 (25.7%)	77 (33.5%)	153 (66.5%)	<0.001	27 (35.1%)	50 (64.9%)	<0.001
ICH score (median)	1 (1-2)	1 (0–2)	3 (2-3)	<0.001	1 (0-1)	2 (1-2)	<0.001
Blood glucose (mg/dl)	162.49 ± 69.05	152.81 ± 61.76	185.58 ± 78.01	<0.001	147.25 ± 57.47	164.06 ± 68.42	0.001
*Imaging data*							
Volume (ml)	21.86 ± 19.20	17.55 ± 15.59	32.45 ± 24.39	<0.001	13.96 ± 10.93	24.72 ± 17.96	<0.001
*Side*							
Right	417 (46.1%)	301 (72.9%)	112 (27.1%)	0.001	203 (67.4%)	98 (32.6%)	0.531
Left	430 (47.5%)	289 (69.5%)	127 (30.5%)		190 (65.7%)	99 (34.3%)	
Midline (cerebellar, brain stem and IVH)	50 (5.5%)	28 (58.3%)	20 (41.7%)		19 (67.9%)	9 (32.1%)	
Bilateral	8 (0.9%)	1 (12.5%)	7 (87.5%)		0 (0%)	1 (100%)	
*Supratentorial hemorrhage*							
Volume	23.45 ± 19.79	43.3 ± 13.89	52.7 ± 21.10	<0.001	40.23 ± 10.31	44.97 ± 15.30	0.021
Volume >30 ml	234 (25.85%)	108 (46.4%)	125 (53.6%)		38 (35.2%)	70 (64.8%)	
*Infratentorial hemorrhage*							
Volume	10.27 ± 6.49	9.07 ± 6.77	12.11 ± 5.72	0.492	8.82 ± 5.95	9.78 ± 8.96	0.038
IVE	425 (47%)	241 (57.8%)	176 (42.2%)	<0.001	137 (56.8%)	104 (43.2%)	<0.001
Hydrocephalus	236 (26.1%)	111 (48.3%)	119 (51.7%)	<0.001	66 (59.5%)	45 (40.5%)	0.095
Hematoma Growth	46 (5.08%)	24 (52.2%)	22 (47.8%)	0.007	12 (50%)	12 (50%)	0.121
*Surgery*							
EVD	56 (6.2%)	23 (41.8%)	32 (58.2%)	<0.001	13 (56.5%)	10 (43.5%)	0.367
Clot evacuation	147 (16.8%)	106 (72.1%)	41 (27.9%)	0.621	36 (34%)	70 (66%)	<0.001

**Table 2:** Distribution of hematoma based on location and their clinic radiological characteristics. GCS—glasgow coma score, ICH—intracerebral hemorrhage, IVE—intraventricular haemorrhage, EVD—external ventricular drain, mRS—modified ranking scale.

Parameter	Supratentorial (780)			Infratentorial (92)		Primary IVH (27)	*p*
	Basal ganglia (478)	Thalamic (202)	Lobar (106)	Cerebellar (61)	Brainstem (31)		
Age	55.90 ± 12.14	62.33 ± 12.15	57.12 ± 13.12	66.74 ± 11.35	55.39 ± 13.46	53.04 ± 13.07	<0.001
*Sex*							
Male	363 (75.9%)	120 (59.4%)	75 (70.8%)	40 (65.5%)	21 (67.7%)	19 (70.4%)	0.001
Female	115 (24.1%)	82 (40.6%)	31 (29.2%)	21 (34.4%)	10 (32.3%)	8 (29.6%)	
GCS (median/ IQR)	11 (8–14)	13 (9–14)	12 (10–14)	13 (8–15)	7 (4–11)	14 (13–15)	<0.001
GCS<8	130 (27.2%)	47 (23.3%)	16 (15.1%)	17 (27.9%)	20 (64.5%)	3 (11.1%)	<0.001
*Glucose*							
Volume	29.10 ± 21.45	11.22 ± 8.05	23.32 ± 16.98	12.21 ± 6.40	6.45 ± 4.81	7.03 ± 3.89	<0.001
IVH	179 (37.4%)	155 (76.7%)	26 (24.5%)	31 (50.8%)	9 (29%)	27 (100%)	<0.001
Hydrocephalus	78 (16.3%)	85 (42.1%)	10 (9.4%)	33 (54.1%)	10 (32.3%)	20 (74.1%)	<0.001
Hematoma growth	35 (7.3%)	7 (3.5%)	3 (2.9%)	1 (1.6%)	0	0	0.036
EVD	14 (2.9%)	20 (9.9%)	2 (1.9%)	6 (9.8%)	2 (6.5%)	3 (11.1%)	<0.001
Clot evacuation	110 (23.01%)	3 (1.48%)	19 (17.9%)	14 (22.9%)	0	0	<0.001
Mortality	152 (31.8%)	55 (27.2%)	20 (18.2%)	19 (31.1%)	17 (54.8%)	3 (11.1%)	0.001
mRS discharge (median)	4 (4-5)	4 (4-5)	4 (3–5)	4 (3–5)	5 (5-6)	2 (2-3)	<0.001
mRS 90 days (median)	4 (3–6)	4 (3–6)	3 (1–4)	3 (2–6)	6 (4–6)	2 (1-2)	<0.001
Good outcome	189 (39.5%)	95 (47%)	66 (62.2%)	34 (55.7%)	6 (19.4%)	22 (81.5%)	<0.001
Poor outcome	128 (26.7%)	46 (22.7%)	18 (17%)	7 (11.4%)	7 (22.6%)	1 (3.7%)	

**Table 3:** ICH score and mortality. ICH—Intracerebral hemorrhage.

ICH score	*N*	Dead	%
0	200	13	7
1	280	44	16
2	208	69	33
3	136	84	62
4	57	54	95
5	4	2	50

**Table 4:** Outcome at discharge and 90 days. mRS—modified ranking scale.

Parameter	Discharge (*n* = 905)	90 days (*n* = 885)
Death	116 (12.81%)	266 (30.1%)
mRS	4 (4–5)	4 (3–6)
Good outcome (mRS 0–3)	196 (21.6%)	412 (66.6%)
Poor outcome (mRS 4–5)	593 (65.5%)	207 (33.4%)
mRS 0	1	27
mRS 1	22	64
mRS 2	40	127
mRS 3	133	194
mRS 4	296	157
mRS 5	297	50
mRS 6	116	266

**Table 5:** Logistic regression of factors influencing mortality and outcome.

Parameter	Mortality			Outcome		
	*p*	Odds ratio	95% C.I.	*p*	Odds ratio	95% C.I.
Age > 70 years	<0.001	4.806	3.064–7.54	0.005	2.125	1.258–3.591
GCS < 8	<0.001	7.684	5.055–11.68	<0.001	3.233	1.836–5.693
Volume > 30 ml	<0.001	2.45	1.626–3.691	<0.001	4.263	2.657–6.841
Intraventricular extension	0.581	1.128	0.736–1.73	0.015	1.69	1.105–2.585
Hydrocephalus	0.014	1.778	1.123–2.815	0.505	0.83	0.479–1.437
Hematoma growth	<0.001	4.104	2.092–8.05	0.145	1.928	0.797–4.662

## Data Availability

The data used to support the findings of this study are available from the corresponding author upon request.
